# Direct growth of freestanding GaN on C-face SiC by HVPE

**DOI:** 10.1038/srep10748

**Published:** 2015-06-02

**Authors:** Yuan Tian, Yongliang Shao, Yongzhong Wu, Xiaopeng Hao, Lei Zhang, Yuanbin Dai, Qin Huo

**Affiliations:** 1 State Key Lab of Crystal Materials, Shandong University, Jinan, 250100, P. R. China

## Abstract

In this work, high quality GaN crystal was successfully grown on C-face 6H-SiC by HVPE using a two steps growth process. Due to the small interaction stress between the GaN and the SiC substrate, the GaN was self-separated from the SiC substrate even with a small thickness of about 100 μm. Moreover, the SiC substrate was excellent without damage after the whole process so that it can be repeatedly used in the GaN growth. Hot phosphoric acid etching (at 240 °C for 30 min) was employed to identify the polarity of the GaN layer. According to the etching results, the obtained layer was Ga-polar GaN. High-resolution X-ray diffraction (HRXRD) and electron backscatter diffraction (EBSD) were done to characterize the quality of the freestanding GaN. The Raman measurements showed that the freestanding GaN film grown on the C-face 6H-SiC was stress-free. The optical properties of the freestanding GaN layer were determined by photoluminescence (PL) spectra.

Recently, GaN-based materials have attracted much interest as a material suitable for use in optoelectronic and electronic devices due to their excellent properties^1^,^2^,^3^. However due to the immature GaN freestanding substrate technology, current commercial GaN-based devices are fabricated by epitaxy onto foreign substrates. The large lattice mismatch and difference in thermal expansion coefficient between the substrate and the layer results in high defect density (10^7^–10^10^/cm^2^), which degrades its optical and electrical properties^4^,^5^,^6^. Thus, freestanding GaN wafers are in urgent need. Hydride vapor phase epitaxy (HVPE) is supposed to be the most promising method to acquire freestanding GaN with the advantages of simple equipment, low cost and quick growth speed^7^. GaN bulk growth using HVPE is started in most cases on c-oriented Al_2_O_3_ substrates. Due to the high lattice mismatch (13.8%), a high dislocation density is formed. In order to improve the crystal quality of GaN film, SiC is considered to be promising substrate material because of small lattice match (3.4%) and similar thermal expansion coefficient. GaN grown on SiC instead of sapphire has better crystal quality and smaller residual stress^8^. Several papers about the direct growth of GaN layers on Si- face SiC substrates by HVPE^9^ have been reported. However, there have been few papers about the direct growth of GaN layers on C-face SiC substrates by HVPE as the growth of high quality GaN on C- face SiC is much more difficult^10^.

In this paper, we successfully obtained high quality, stress-free GaN layer which directly self-separated from the SiC substrate. This method avoids ex situ processing to separate GaN layer from substrate before or after the growth. This is very different from the ones reported before such as mechanical lapping of sapphire^11^, laser lift-off^12^, chemical etching^13^, void assisted separation^14^ and ELOG^15^. Moreover, due to the small interaction stress between GaN and the SiC substrate, the SiC substrate was excellent without damage after the whole process so that it can be repeatedly used in the GaN growth.

## Experimental

The growth was done in a home-built vertical HVPE reactor at atmospheric pressure. The precursors and carrier gases were delivered from the bottom. GaCl, NH_3_ and N_2_ were used as gallium source, nitrogen source and carrier gas, respectively. The C-face 6H-SiC substrate was employed in the subsequent HVPE growth. HCl gas reacted with liquid Ga at 500-800 °C to form GaCl, which was then transported to the template where it reacted with NH_3_ to form GaN.

**Figure 1:**
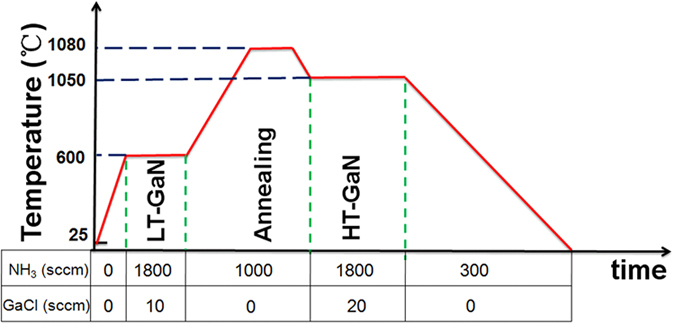
Schematic diagram of the growth process.

Figure 1 shows the different growth steps. First, the substrate was heated to 600 °C for deposition of low temperature GaN (LT-GaN) buffer layer, which was grown with 1800 sccm of NH_3_ and 10 sccm of GaCl for 30 min. Thereafter, the temperature was increased to 1080 °C and kept constant for 10 min. The layer was annealed in this step in presence of NH_3_ to improve the crystallinity. Then, thick high temperature GaN (HT-GaN) was grown at 1050 °C with 1800 sccm of NH_3_ and 20 sccm of GaCl. Finally, the temperature was cooled down with 300 sccm of NH_3_.

The morphologies of GaN were investigated by a field emission scanning electron microscope (FE-SEM, Hitachi S-4800). A cross-sectional EBSD measurement was carried out by an EBSD system (Oxford Instruments INCA Crystal EBSD system, Nordlys EBSD Detector and HKL CHANNEL5 software) in the FE-SEM. Structural characterization of LT-GaN buffer was carried out with a Burker AXS X-ray system using Cu-Kα radiation. The Raman spectroscopy (LabRAM HR system of Horiba Jobin Yvon) at room temperature using a 532 nm solid state laser as the excitation source was employed to identify the stress of the freestanding GaN. The accuracy during the Raman measurements was 0.5 cm^−1^ with a spot size of approximately 2.0 mm. High-resolution X-ray diffraction (HRXRD) characterizations were performed on a Bruker D8 Discover High Resolution X-Ray Diffractometer using symmetrical (002) and asymmetrical (102) reflections, which provided a resolution of about 13 arcsec and a slit width of 0.6 mm. The optical property was determined by room temperature photoluminescence (PL) measurements with a 325 nm He–Cd laser as the excitation source.

## Results and discussion

**Figure 2:**
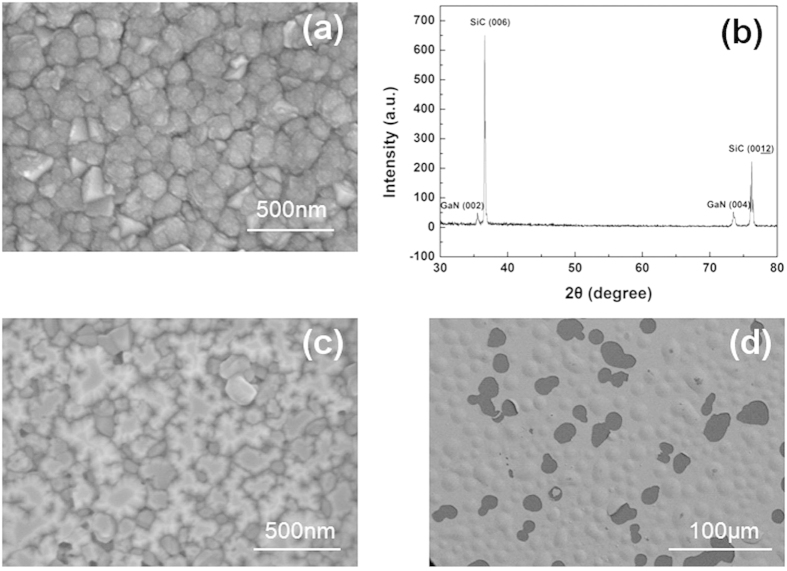
SEM images of LT-GaN buffer before annealing (**a**), after annealing (**c**,**d**) and 2*θ − ω* XRD diffraction pattern of LT-GaN buffer (b).

The morphology of LT-GaN buffer before annealing with randomly oriented three dimensional GaN islands formed at onset of nucleation is shown in Fig. 2a. The image demonstrates a clear Volmer-Weber island growth mode because of the poor surface wetting between GaN and SiC^16^,^17^. The subsequent annealing step recrystallizes the layer. After annealing, the morphology changes and some flat regions appear as shown in Fig. 2c. Figure 2b shows 2*θ* *−* *ω* XRD diffraction pattern of LT-GaN buffer on SiC before annealing. From Fig. 2b, the LT-GaN buffer studied in this work has a single crystalline structure. The position of the peaks corresponds to wurtzite GaN and 6H-SiC, with the epitaxial relationship (0001) GaN//(0001) 6H-SiC.

**Figure 3:**
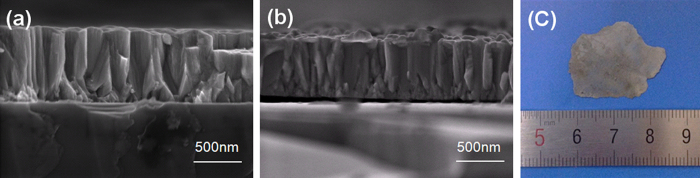
SEM images of cross-section of LT-GaN buffer before annealing (**a**) and after annealing (**b**), backside of freestanding GaN (**c**).

After cooling down process, a mirror-like GaN layer with a thickness of about 100 μm and area of about 4 cm^2^ was spontaneously separated from SiC substrate. The picture of freestanding GaN is shown in Fig. 3c. The separation occurred at the interface of the buffer and the SiC. The LT-GaN buffer and the annealing process are responsible for the separation of the GaN and the SiC. At high temperature, GaN is decomposed into liquid gallium and nitrogen gas. The threshold temperature for the thermal decomposition of GaN is known to be 830 °C^18^ and the decomposition rate rises with temperature^19^. LT-GaN buffer was grown on 6H-SiC directly. The crystallographic orientation relationship between GaN and 6H-SiC is shown as follows:


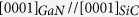



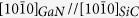


Though the LT-GaN buffer studied in this work has a single crystalline structure, the crystal quality is poor which can be seen from Fig. 2b. This made the decomposition much easier. LT-GaN buffer before annealing was not separated from SiC. Figure 3a shows the cross-section of LT-GaN buffer before annealing. There are many small voids at the interface between LT-GaN buffer and SiC. The voids at the interface are confined, as a result, the supply of source materials for GaN, i.e., NH_3_ and GaCl, is virtually stopped. During annealing process, decomposition of GaN in the confined space begins as a result of the high temperature. The decomposition is significant on the N-face of the islands, and buffer layer separated from SiC. Fig. 3b shows the cross-section of the LT-GaN buffer after annealing. The buffer is separated with the substrate though the buffer is very thin (about 500 nm) which indicates a very small interaction force between GaN and the substrate. This can also be seen from the bird’s-eye view of the LT-GaN buffer after annealing with many swell and drop off. (Figure 2d)

**Figure 4:**
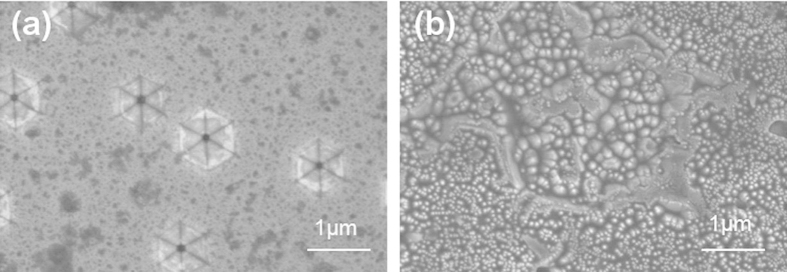
SEM images of the surface morphology and back surface morphology of freestanding GaN after hot phosphoric acid etching.

The freestanding GaN was etched in phosphoric acid at 240 °C for 30 min.The morphologies of the surface (Fig. 4a) and the back surface (Fig. 4b) after etching were shown in Fig. 4. GaN polarity can be identified after hot phosphoric acid etching since the etching rate and the resulting surface morphology is polarity dependent. Only N-polar GaN epilayers can be etched in phosphoric acid at 100 °C^20^. When the etching temperature is higher than 220 °C, the N-polar GaN films etched quickly, with either a drastic change in the surface morphology or complete film removal while only hexagonal etch pits associated with defects formed on Ga-polar films, leaving the defect-free GaN areas intact and the morphology unchanged^21^,^22^,^23^,^24^,^25^,^26^. According to Fig. 4, the GaN layer grown on C face 6H-SiC in this study is Ga-polar GaN.

Raman spectroscopy has been widely used to identify the property of III-nitrides. In GaN, the E_2_ (high) phonon of Raman spectroscopy is used to characterize the in plane stress state of the GaN epilayer. The stress can be calculated by equation (1)^27^:


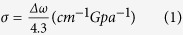


**Figure 5:**
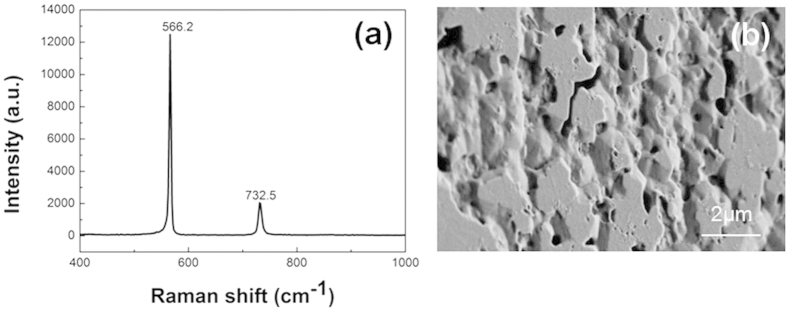
Raman spectroscopy of freestanding GaN (**a**) and SEM image of the back surface morphology (**b**).

Where σ is the biaxial stress and △ω is the E_2_ phonon peak shift. The E_2_ phonon of stress-free GaN is believed to be 566.2 cm^−1^ 28. Figure 5a depicts the Raman spectra of the freestanding GaN grown on C face 6H-SiC. The position of E_2_ (high) phonon is at 566.2 cm^−1^, which indicates that the freestanding GaN grown on C face 6H-SiC is stress-free. Figure 5b shows the the back surface morphology of freestanding GaN. The decomposed area is more than 50%, which explains the stress state of the freestanding GaN.

**Figure 6:**
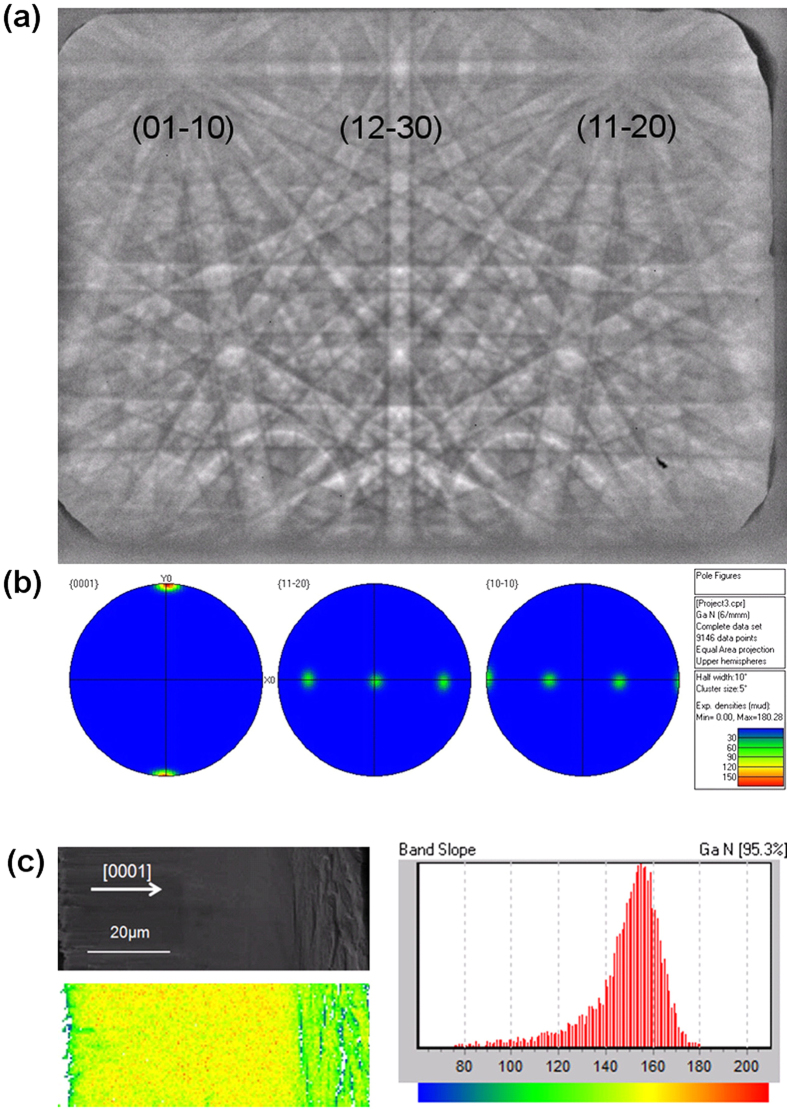
EBSD Kikuchi patterns (**a**), pole figures (**b**) and band slope (**c**) detected in cross-sectional GaN.

The electron backscatter diffraction (EBSD) technique in a scanning electron microscope (SEM) is an effective technique for studying the crystal phase, orientation, and lattice strain variation that are typical in semiconducting materials^29^,^30^. This technique allows us to probe the mechanism of the crystal quality improvement at the microstructural level with high spatial resolution and good strain sensitivity^31^,^32^,^33^. The Kikuchi patterns in EBSD is widely used to identify the crystallographic orientation of crystalline by conform the Miller index of the surface. Figure 6a showed the Kikuchi patterns in cross-sectional surface of freestanding GaN, the patterns were clear which means that the surface was flat and the crystal structure of GaN was perfect. The result indicates that the GaN cross-sectional surface is (11-20) plane.

The EBSD mapping measurement was carried out at the cross-section of freestanding GaN. The polar figures of this section (Fig. 6b) were calculated by the HKL CHANNEL5 software depending on the mapping results, which confirm that the surface detected is (11-20) plane. The band slope (BS) was a measure of the quality of the original electronic backscattering patterns (EBSP) used in the indexing procedure. BS value is derived from the mean gradient of detected peaks in the Hough transform, and is a measure of the sharpness of bands, with higher values representing better EBSP quality. The BS value is strain sensitive, especially in measurement of single crystals. Meanwhile, it reflects local dislocation concentrations. Figure 6c shows the BS from EBSD mapping data for cross-sectional surface of freestanding GaN. The BS value is small near the interface and increases when the distance to the interface becomes large. This predicts a decrease of strain and dislocation concentration upon growth. To be clear, the BS varies with surface polish or damage^34^, so the BS valve increases acutely near the surface corresponding to the rough area.

**Figure 7:**
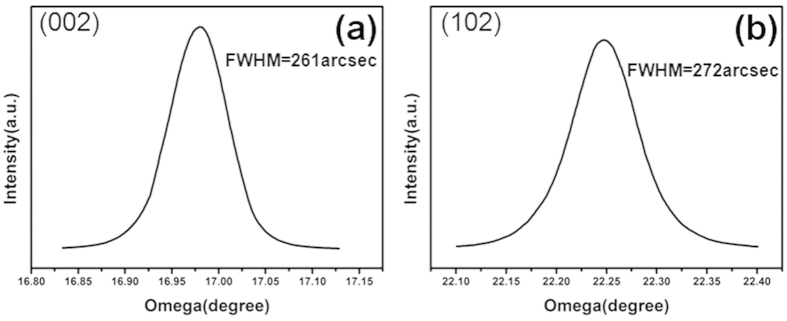
HRXRD rocking curves of the freestanding GaN films: (**a**) (002) ω-scans and (**b**) (102) ω-scans.

Figure 7 shows the ω-scans spectra from the symmetric (002) and asymmetric (102) planes of the freestanding GaN layers. HRXRD characterizations were performed to examine the misorientation between sub-grains, which mitigated by generation of dislocations. mosaic tilt reflects screw and mixed type TD density while twist indicates edge and mixed TD density. The full width at half maximum (FWHM) on the (002) reflection probes tilt which represents misorientation of sub-grains out-of-plane. The FWHM on the (102) reflection is sensitive to both tilt and twist. Thus, the FWHM of the (002) peak is usually used to evaluate the screw or mixed TDs density while the (102) peak is sensitive to all TDs. The FWHM of (002) peak is 261 arcsec (Fig. 7a) and the FWHM of (102) peak is 272 arcsec (Fig. 7b). The dislocation density can be calculated from equation (2)^35^:


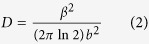


Where β is the FWHM and b is the Burgers vector. The calculated TD density in GaN layer is 1.17 E + 08 cm^−2^.

**Figure 8:**
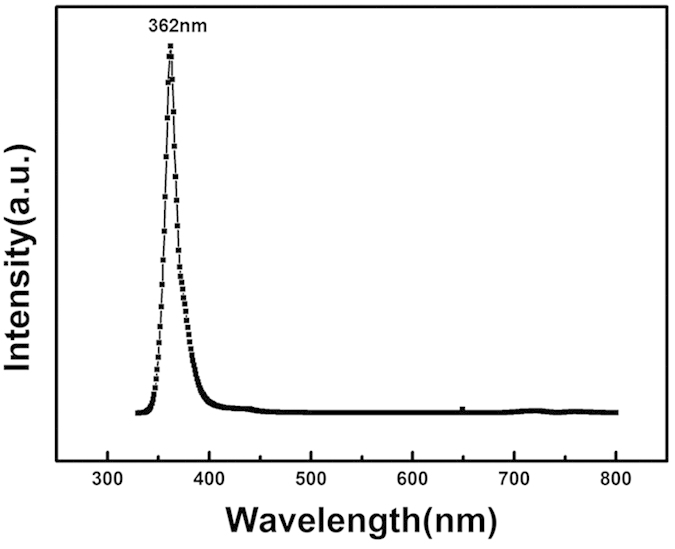
PL spectra of the freestanding GaN.

Figure 8 shows the room temperature PL spectra of the freestanding GaN grown on C face 6H-SiC. A strong band edge emission of GaN is found at 362 nm (3.425 eV), which confirms that the GaN layer is a hexagonal phase rather than cubic phase (excitonic emission around 3.2 eV).

The TDs in the GaN film play the role of non-radiative recombination centers to deteriorate the luminescence efficiency^36^. The strong band edge emission demonstrates that the optical quality of the HVPE GaN layer is good and the TDs density is low. The FWHM of the band-edge emission is 123 meV. The very weak yellow luminescence band at 500–600 nm implies a low density of native defects, such as vacancies, interstitials and anti-sites in the sample^37^,^38^,^39^.

For a semiconductor, the band gap energy is changed by the stress applied to the layer. The band gap energy of GaN at room temperature under c-plane was reported as equation (3)^40^:


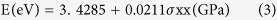


According equation (3), the strain of the freestanding GaN is approach to zero, which quite fits in with the result of the Raman spectroscopy.

## Conclusion

The direct growth of GaN on the C-face 6H-SiC by HVPE has been demonstrated. The GaN was self-seperated from the 6H-SiC which can save both time and money to separate GaN layer and substrate. Moreover, the SiC substrate was excellent without damage after the whole process so that it can be repeatedly used in the GaN growth. The self-separation was attributed to the LT-GaN buffer and the annealing process. According to the hot phosphoric acid etching results, the obtained layer was Ga-polar GaN. According to the HRXRD results, the FWHM of (002) peak was 261 arcsec and the The FWHM of (102) peak was 272 arcsec. EBSD carried out at the cross-section of the freestanding GaN proved a high crystal quality and a decrease of strain and dislocation concentration upon growth. The Raman measurements and PL spectra showed that the freestanding GaN film grown on the C-face 6H-SiC was stress-free. The PL spectra also indicated good optical quality of the freestanding GaN layer.

## Additional Information

**How to cite this article**: Tian, Y. *et al*. Direct growth of freestanding GaN on C-face SiC by HVPE. *Sci. Rep.* **5**, 10748; doi: 10.1038/srep10748 (2015).

## References

[b1] Jia H., Guo L., Wang W. & Chen H. Recent progress in GaN‐based light‐emitting diodes. Adv. Mater. 21, 4641–4646 (2009).

[b2] Nakamura S., Mukai T. & Senoh M. Candela‐class high‐brightness InGaN/AlGaN double‐heterostructure blue‐light‐emitting diodes. Appl. Phys. Lett. 64, 1687–1689 (1994).

[b3] Oshima Y., Eri T., Shibata M., Sunakawa H. & Usui A. Fabrication of Freestanding GaN Wafers by Hydride Vapor‐Phase Epitaxy with Void‐Assisted Separation. Phys. Status Solidi A 194, 554–558 (2002).

[b4] Morkoç H. Handbook of Nitride Semiconductors and Devices (WILEY-VCH Verlag GmbH & Co. KGaA, Weinheim, 2008).

[b5] Wang F. C., Cheng C. L., Chen Y. F., Huang C. F. & Yang C. C. Residual thermal strain in thick GaN epifilms revealed by cross-sectional Raman scattering and cathodoluminescence spectra. Semicond. Sci. Tech. 22, 896 (2007).

[b6] Cho S. I., Chang K. & Kwon M. S. Strain analysis of a GaN epilayer grown on a c-plane sapphire substrate with different growth times. J. Mater. Sci. 42, 3569–3572 (2007).

[b7] Dai Y. *et al.* Influence of V/III ratio on stress control in GaN grown on different templates by hydride vapour phase epitaxy. RSC Adv. 4, 21504–21509 (2014).

[b8] Zhang L. *et al.* Comparison of the strain of GaN films grown on MOCVD-GaN/Al_2_O_3_ and MOCVD-GaN/SiC samples by HVPE growth. J. Cryst. Growth 335, 62–66 (2011).

[b9] Melnik Y. V., Nikitina I. P., Nikolaev A. E. & Dmitriev V. A. Structural properties of GaN grown on SiC substrates by hydride vapor phase epitaxy. Diam. Relat. Mater. 6, 1532–1535 (1997).

[b10] Keller S. *et al.* Effect of the Nucleation Conditions on the Polarity of AlN and GaN Films Grown on C-face 6H–SiC. Jpn. J. Appl. Phys. 45, L322–L325 (2006).

[b11] Kim H. M., Oh J. E. & Kang T. W. Preparation of large area free-standing GaN substrates by HVPE using mechanical polishing liftoff method. Mater. Lett. 47, 276–280 (2001).

[b12] Kelly M. K. *et al.* Large free-standing GaN substrates by hydride vapor phase epitaxy and laser-induced lift off. Jpn. J. Appl. Phys. 38, L217 (1999).

[b13] Zhang L. *et al.* Improvement of crystal quality HVPE grown GaN on an H_3_ PO_4_ etched template. CrystEngComm 13, 5001–5004 (2011).

[b14] Oshima Y. *et al.* Preparation of freestanding GaN wafers by hydride vapor phase epitaxy with void-assisted separation. Jpn. J. Appl. Phys. 42, L1 (2003).

[b15] Gogova D. *et al.* Strain-free bulk-like GaN grown by hydride-vapor-phase-epitaxy on two-step epitaxial lateral overgrown GaN template. J. Appl. Phys. 96, 799–806 (2004).

[b16] Moran B. *et al.* Structural and morphological evolution of GaN grown by metalorganic chemical vapor deposition on SiC substrates using an AlN initial layer. J. Cryst. Growth 273, 38–47 (2004).

[b17] Fini P. *et al.* The effect of growth environment on the morphological and extended defect evolution in GaN grown by metalorganic chemical vapor deposition. Jpn. J. Appl. Phys. 37, 4460–4466 (1998).

[b18] Ambacher O. *et al.* Thermal stability and desorption of Group III nitrides prepared by metal organic chemical vapor deposition. J. Vac. Sci. Technol. B 14, 3532–3542 (1996).

[b19] Rebey A., Boufaden T. & El Jani B. *In situ* optical monitoring of the decomposition of GaN thin films. J. Cryst. Growth 203, 12–17 (1999).

[b20] Lee C. *et al.* Selective chemical etch of gallium nitride by phosphoric acid. J. Vac. Sci. Technol. A 30, 040602 (2012).

[b21] Huang D. *et al.* Dependence of GaN polarity on the parameters of the buffer layer grown by molecular beam epitaxy. Appl. Phys. Lett. 78, 4145–4147 (2001).

[b22] Visconti P. *et al.* Dislocation density in GaN determined by photoelectrochemical and hot-wet etching. Appl. Phys. Lett. 77, 3532–3534 (2000).

[b23] Visconti P. *et al.* Investigation of defects and polarity in GaN using hot wet etching, atomic force and transmission electron microscopy and convergent beam electron diffraction. Phys. Status Solidi B 228, 513–517 (2001).

[b24] Visconti P. *et al.* Rapid delineation of extended defects in GaN and a novel method for their reduction. Phys. Status Solidi A 190, 5–14 (2002).

[b25] Visconti P. *et al.* Investigation of defects and surface polarity in GaN using hot wet etching together with microscopy and diffraction techniques. Mat. Sci. Eng. B 93, 229–233 (2002).

[b26] Onabe K. [Blue Laser and Light Emitting Diodes II] International Symposium on Blue Laser and Light Emitting Diodes II [ Shimizu M., Suzuki A., Watanabe M., Shirakashi J., Balakrishan K., Okumura H. (eds.)] (Chiba, Japan, 1998).

[b27] Tripathy S., Chua S. J., Chen P. & Miao Z. L. Micro-Raman investigation of strain in GaN and Al_x_Ga_1− x_N/GaN heterostructures grown on Si (111). J. Appl. Phys. 92, 3503–3510 (2002).

[b28] Kisielowski C. *et al.* Strain-related phenomena in GaN thin films. Phys. Rev. B 54, 17745 (1996). 10.1103/physrevb.54.17745 9985904

[b29] Shao Y. *et al.* Large Area Stress Distribution in Crystalline Materials Calculated from Lattice Deformation Identified by Electron Backscatter Diffraction. Sci Rep. 4, 5934 (2014). 25091314 10.1038/srep05934 PMC4121609

[b30] Luo J. F. *et al.* EBSD measurements of elastic strain fields in a GaN/sapphire structure. Microelectron. Reliab. 46, 178–182 (2006).

[b31] Stanford N., Dunne D. & Ferry M. Deformation and annealing of (011)[01-1] oriented Al single crystals. Acta Mater 51, 665 (2003).

[b32] Wilkinson A.J. & Hirsh P.B. Electron diffraction based techniques in scanning electron microscopy of bulk materials. Micron 28, 279–308 (1997).

[b33] Shao Y. *et al.* EBSD crystallographic orientation research on strain distribution in hydride vapor phase epitaxy GaN grown on patterned substrate. CrystEngComm 15, 7965–7969 (2013).

[b34] Wright S. I. & Nowell M. M. EBSD image quality mapping. Microsc. Microanal. 12, 72–84 (2006). 17481343 10.1017/S1431927606060090

[b35] Chaudhuri J., Ng M. H., Koleske D. D., Wickenden A. E. & Henry R. L. High resolution X-ray diffraction and X-ray topography study of GaN on sapphire. Mater. Sci. Eng. B 64, 99–106 (1999).

[b36] Choi Y. S. *et al.* Effects of dislocations on the luminescence of GaN/InGaN multi-quantum-well light-emitting-diode layers. Mater. Lett. 58, 2614–2617 (2004).

[b37] Bogusl P., Briggs E. L. & Bernholc J. Native defects in gallium nitride. Phys. Rev. B 51, 17255 (1995). 10.1103/physrevb.51.17255 9978750

[b38] Lyons J. L., Janotti A. & Van de Walle C. G. Carbon impurities and the yellow luminescence in GaN. Appl. Phys. Lett. 97, 152108 (2010).

[b39] Xu F. *et al.* Different origins of the yellow luminescence in as-grown high-resistance GaN and unintentional-doped GaN films. J. Appl. Phys. 107, 023528 (2010).

[b40] Zhao D. G., Xu S. J., Xie M. H., Tong S. Y. & Yang H. Stress and its effect on optical properties of GaN epilayers grown on Si (111), 6H-SiC (0001), and c-plane sapphire. Appl. Phys. Lett. 83, 677–679 (2003).

